# Potassium Transporter *LrKUP8* Is Essential for K^+^ Preservation in *Lycium ruthenicum*, A Salt-Resistant Desert Shrub

**DOI:** 10.3390/genes10080600

**Published:** 2019-08-09

**Authors:** Fengbin Dai, Aijia Li, Shupei Rao, Jinhuan Chen

**Affiliations:** 1 College of Biological Sciences and technology, Beijing Forestry University, 35 Qinghua East Road, Beijing 100083, China; 2 National Engineering Laboratory for Tree Breeding, Beijing Forestry University, 35 Qinghua East Road, Beijing 100083, China

**Keywords:** callus, *Lycium ruthenicum*, salt stress, K^+^/Na^+^ homeostasis

## Abstract

Salt stress is a major constraint for many crops and trees. A wild species of Goji named *Lycium ruthenicum* is an important economic halophyte in China and has an extremely high tolerance to salinity. *L. ruthenicum* grows in saline soil and is known as a potash-rich species. However, its salt adaptation strategies and ion balance mechanism remains poorly understood. Potassium (K^+^) is one of the essential macronutrients for plant growth and development. In this study, a putative salt stress-responsive gene encoding a HAK (high-affinity K^+^)/KUP (K^+^ uptake)/KT (K^+^ transporter) transporter was cloned and designated as *LrKUP8*. This gene belongs to the cluster II group of the KT/HAK/KUP family. The expression of *LrKUP8* was strongly induced under high NaCl concentrations. The OE-*LrKUP8* calli grew significantly better than the vector control calli under salt stress conditions. Further estimation by ion content and micro-electrode ion flux indicated a relative weaker K^+^ efflux in the OE-*LrKUP8* calli than in the control. Thus, a key gene involved in K^+^ uptake under salt condition was functionally characterized using a newly established *L. ruthenicum* callus transformation system. The importance of K^+^ regulation in *L. ruthenicum* under salt tolerance was highlighted.

## 1. Introduction

Soil salinization is a severe global environmental stress that limits the growth and development of trees and crops. Over 800 million hectares of the world’s arable land is adversely affected by salinity [1]. Halophytes are plants that tolerate high salt concentrations that kill 99% of other species [2]. These plants can complete an entire life cycle in a salt concentration of at least 200 mM NaCl, either in controlled conditions or the natural environment [3]. Halophytes draw considerable attention [4,5,6], and many studies focused on the ability of maintaining an optimal K^+^/Na^+^ ratio in the cytosol [7,8,9,10].

K^+^ and Na^+^ have similar chemical properties and content ratio in non-saline soils [11]. K^+^ is an essential nutrient required for plant growth and development, and plays important roles in protein synthesis and the maintenance of cytoplasmic pH and transmembrane voltage gradients [12]. Low Na^+^ and high K^+^ in the cytoplasm are important in upholding several enzymatic processes [13]. An extremely high Na^+^ as a toxic ion in salinized soil results in ion-induced injury of plant cells. Thus, precisely controlling Na^+^/K^+^ selective accumulation is needed to maintain cellular cation homeostasis [14].

For K^+^ acquisition and distribution, plants have two K^+^ transport systems: K^+^ channels and K^+^ transporters [15]. The four families of K^+^ transport systems are shaker channels, K^+^ channel outward-rectifier (KCO) channels, high-affinity K^+^ transporter (HKT) transporters, and KT/KUP/HAK transporters [11,16]. Genes from the KT/KUP/HAK family can be divided into four clusters (I–IV) [17,18]. Previous studies on *Arabidopsis thaliana* identified AtHAK5, a high-affinity K^+^ transporter, as the major contributor to K^+^ uptake from low-K^+^ solutions under saline conditions [19,20]. Many genes in cluster I were studied, and all these transporters can mediate high-affinity K uptake [21,22]. Cluster II members of *KT/KUP/HAK* have greatly diverse functions [23]. *AtKUP1* mediates high-and low-affinity K uptake [24]. *OsHAK2* exhibits higher Na than K transport activities [21]. Arabidopsis *kup4* mutant results in tiny root hairs due to impaired auxin transport, whereas *kup2* mutant decreases the cell expansion in the shoot [25,26]. All these studies indicated that KT/KUP/HAK transporters play important roles in K^+^ acquisition, redistribution, and homeostasis. However, the physiological functions in terms of K acquisition under salt stresses focused only on several genes that are mainly from model plants. Many individual members of KT/KUP/HAK transporters are not yet characterized in plants, especially in halophytes.

*L. ruthenicum* is a wild Goji species that recently attracted attention due to its nutritional and medical values [27]. This plant is described in the Tibetan medical classics Jing Zhu Ben Cao and Si Bu Yi Dian as a treatment for heart disease, abnormal menstruation, and menopause [28]. Studies on this species focused on determining its active pharmaceutical ingredients and the pharmacological activities of its chemical composition, extracting its active constituents, and developing seedling-breeding techniques [29,30,31,32]. *L. ruthenicum* exhibits extremely high salt tolerance [33], which can alleviate the degree of soil salinity–alkalinity and is crucial for the restoration of desert ecosystems [27,34]. However, the mechanisms for maintaining K^+^/Na^+^ homeostasis in *L. ruthenicum* are still unknown.

Here, we identified a K^+^ transporter from *L. ruthenicum* according to our transcriptomic data screening. This K^+^ transporter belongs to cluster II of the KT/KUP/HAK family. Functional properties and the expression pattern of this gene suggest its involvement in salt stress response. We studied its ion accumulation mechanism that elicits salt resistance by using a callus system. This work helps elucidate the factors underlying *L. ruthenicum* survival in saline areas and the maintenance of a high K content in highly saline soil.

## 2. Materials and Methods

### 2.1. Plant Material for Ion Content Analysis

*L. ruthenicum* is an economic shrub widely distributed in the northwest arid region of China. During our survey in the Qinghai, Xinjiang Provinces, and Inner Mongolia, we found that *L. ruthenicum* could grow with Na^+^ from 100 mM to 450 mM during control experiments or in natural conditions. From 21 May 2014, we planted 30 *L. ruthenicum* in pots of 60-cm diameter in Qinghai Province and divided the plants into four groups. Natural soil with less than 150 mM (90–122 mM) Na^+^ was first used in planting. On 10 July 2015, 20 pots with similar growth status were selected. A salt control experiment was then performed using 200 mM NaCl solution until the soil Na^+^ reached 200, 300, or 400 mM with errors of approximately 40 mM. At different soil salinity gradients, we analyzed the contents of K^+^ in specific tissues of these *L. ruthenicum* shrubs on 25 August 2017. The trees grown in natural soil were used as controls.

### 2.2. Determination of Na^+^ and K^+^ Contents

Dry weights (DWs) of the samples were determined after drying at 80 °C for 72 h in an oven. The dried samples were then powdered in mortars. Before the Na^+^ and K^+^ concentrations were confirmed, the powdered samples were dissolved by adding 5 mL of concentrated sulfuric acid and 2 mL of 30% H_2_O_2_ on an anti-boiling furnace at 350 °C until the liquid became colorless. An atomic absorption spectrophotometer was used to measure the Na^+^ and K^+^ contents [35]. Three replicate measurements were carried out per treatment.

### 2.3. Callus Induction, Selection, and Maintenance

Wild *L. ruthenicum* seeds were collected from Shaya country town of Xinjiang Province, China in August 2016. The seeds were washed with sodium hypochlorite and distilled water and then germinated in petri plates for five days. Bibulous paper saturated with sterile water was required in the petri plates, which were placed in a dark environment at 25 °C. After leaf emergence, the seedlings were cultured in a Murashige and Skoog (MS) medium with a pH of 6.0. Embryogenic calli initiated from leaves were grown on MS and vitamins supplemented with 30 g·L^−1^ sucrose, 0.6 g·L^−1^ 6-benzylaminopurine (6-BA), and 0.4 g·L^−1^ 2,4-dichlorophenoxyacetic acid (2,4-D), solidified with 7 g·L^−1^ agar.

As *L. ruthenicum* is a self-incompatible open-pollinated species, calli formed on individual seedlings were maintained as independent genotypic lines. Then, 0.5 g of loose and fragile light-yellow embryogenic calli from leaves of each genotypic line were selected and inoculated into the above subculture medium. The fresh embryogenic calli were weighed after being cultured for 30 days and temperature of 28 °C in the dark. The regeneration ability of the calli from each line was calculated as follows: (total fresh weight − initial fresh weight)/initial fresh weight. After the cell line with the highest regeneration ability was selected, the callus induction and subculture were performed every four weeks at 28 °C in the dark to maintain the cell line.

### 2.4. Isolation of Putative Key Genes Responsible for K^+^ /Na^+^ Homeostasis

By screening our previous RNA-sequencing data (No. SRP059046 of the SRA database) [36], we found a potassium transporter that was highly expressed after salt treatment. Its full length was amplified from young *L. ruthenicum* seedlings by a PCR method with specific primers (Table S1, Supplementary Materials). After sequencing, the transporter was designated as K^+^ uptake permease 8 (*LrKUP8*). The protein sequences encoded by the *LrKUP8* and their homologous genes were obstained from *Oryza sativa*, *A. thaliana*, and *Populus* were obtained from the NCBI (http://www.ncbi.nlm.nih.gov/), TAIR (https://www.arabidopsis.org/), and Phytozome (https://phytozome.jgi.doe.gov/pz/portal.html) databases. The sequence alignment was used the DNAMAN 6.0 program, by which the identical and similar base could be easily identified from the output file. Phylogenetic analyses were completed using the MEGA 5.1 program with 1000 bootstrap replicates after aligning sequences with ClustalW (opening = 10, extension = 0.2).

### 2.5. Subcellular Localization of LrKUP8

For subcellular localization, the complete open reading frame (ORF) of *LrKUP8* was amplified via PCR and inserted into a vector pBI121 to generate a fusion construct (35S:*LrKUP8*-GFP). The fusion construct and the control vector were transiently transfected into epidermal cells of *Nicotiana Benthamiana* according to the method of Voinnet et al. [37]. The transformed cells were subsequently observed by laser confocal fluorescence microscopy via a Leica TCS SP8 instrument (Weztlar, Germany).

### 2.6. Gene Expression Analysis by Quantitative Real-Time PCR

Quantitative real-time PCR (qRT-PCR) analysis was used to study tissue specific expression patterns and salt stress-inducing patterns. Total RNA was extracted using the cetyltrimethylammonium bromide method [38]. For tissue-specific analysis, three-year-old *L. ruthenicum* trees were used. Total RNA was extracted from the tissues of the root, shoot, and new and old leaves. For salt-inducible analysis, we extracted total RNA from 300 mM and 400 mM NaCl-treated calli according to a time-course of 1, 3, 6, and 12 h, and non-salt treated calli were used as the control. We tested RNA integrity and quality by gel electrophoresis and by measuring 260/230 and 260/280 absorbance ratios with a NanoDrop 2000 Spectrophotometer (Thermo Fisher Scientific, Waltham, MA, USA). According to the instruction book of the producer (Qiagen, Duesseldorf, Germany), 2 μg of total RNA was used for the reverse-transcription reaction with a Tiangen FastQuant RT Kit (with gDNase, TIANGEN Biotech Co., Beijing, China). All qRT-PCR reactions were completed using ABI SYBR Green PCR Master Mix (Applied Biosystems, Waltham, MA, USA). Relative expression ratios were normalized to the housekeeping gene *Actin1* [39]. All primers used are listed in Table S1 (Supplementary Materials). Details of the qRT-PCR are as follows: 2 μg of total RNA was reverse-transcribed with SuperScriptII (Invitrogen, Carlsbad, CA, USA) using Oligo (d_T_) primers (Invitrogen) in a total volume of 25 μL. The reverse-transcription product was then diluted with 25 μL of sterilized water to a final volume of 50 μL. Then, 1 μL of the diluted solution was used as the template in the qRT-PCR experiments. 20 µL reaction system contained 10 µL SYBR Premix ExTaq, 0.02 µL ROX reference dye, 0.4 µL forward primer, 0.4 µL reverse primer, 1 µL diluted cDNA and 8.18 µL ddH_2_O. The thermal cycle program used was as follows: 94 °C for 20 s, 40 cycles at 94 °C for 20 s, 60 °C for 20 s and 72 °C for 30 s. The melt curve was produced between 60 °C and 95 °C with an increments of 0.5 °C every 5 s. The relative expression of *LrKUP8* in different tissues was analyzed using the 2^−ΔCT^ method and the real-time PCR data under salt treatments were analyzed using the 2^−ΔΔCT^ method [40].

### 2.7. Agrobacterium-Mediated Transformation of L. ruthenicum Calli

We inserted the pBI121*-LrKUP8-GFP* recombinant vector into *A. tumefaciens* strain GV3101. The *Agrobacterium* strains were stored on a yeast extract peptone (YEP) medium with 50 mg·L^−1^ kanamycin sulfate and 50 mg·L^−1^ gentamicin. The culture was incubated overnight at 28 °C until the optical density (OD)600 reached 0.6–0.8. Then, 200 μL of *Agrobacterium* cells were re-cultured in 200 mL of liquid YEP medium under the same conditions. There were no selective antibiotics in this liquid YEP medium. When the OD600 reached 0.5, the liquids were used to transform callus cells. The cells were firstly co-cultured on solid MS medium without antibiotics in darkness at 24 °C for 72 h and then transferred to a screening medium supplemented with 50 mg·L^−1^ kanamycin and 300 mg·L^−1^ cefalothin sodium. The selection period lasted for approximately 30 days. Transgenic callus cells were selected and subcultured every four weeks on a screening medium. For validation of gene presence, total DNA was extracted from putative transgenic calli, the wild-type control, and the vector control. All DNA samples were subjected to PCR using the PCR Kit (TIANGEN, China) with primer sets to amplify the *NptII*, *GFP*, and *LrKUP8-GFP* genes. Table S1 (Supplementary Materials) provides a list of primers used.

### 2.8. Observation of GFP Fluorescence

The transformed callus was placed in a screening medium and cultured for 20 days under dark conditions before this experiment. Before imaging, the calli were selected from the solid plates and placed in 1/2 MS liquid medium to obtain the relatively dispersed callus cell. GFP imaging was achieved with a Leica SP8 confocal laser scanning microscope (Leica, Weztlar, Germany). Fluorescent protein measurements were conducted with excitation at 488 nm and emission at 525 ± 25 nm for GFP. The image of bright field channel was also acquired as a background.

### 2.9. Determination of Callus Growth under Salt Stress

To test the performance of *LrKUP8*, the pBI121-GFP control and *LrKUP8* overexpressing calli (OE-*LrKUP8*, fresh weight < 0.1 g) were shifted to an MS medium with 0, 100, 200, and 300 mM NaCl with 50 mM kanamycin. After 50 days, calli from the four culture dishes were observed and the fresh weight was determined. The treatment was performed three times.

### 2.10. Ion Contents

At 50 days after the calli were transferred to the 300 mM salinized culture medium, the DWs of the separated calli were determined after drying at 80 °C for 72 h in an oven to a constant weight. The Na^+^ and K^+^ contents were measured using the method described above.

### 2.11. Ion Flux

We measured net fluxes of K^+^ by a non-invasive micro-test technique (NMT, BIO-001A, Younger USA Sci. & Tech. Corp., Amherst, MA, USA) at Xuyue Science and Technology Co., Ltd., Beijing, China [41]. The non-invasive micro-test technique originated from the Woods Hole Marine Biological Laboratory in the United States. In 1995, the Woods Hole Institute firstly modified the vibration probe device to make it capable of ion current [42]. Before use, the calli were grown for 12 h in two types of callus regeneration medium with or without 300 mM NaCl. The samples were rinsed with redistilled water to decrease the effect of salt release on flux recording. The K^+^ ion-selective microelectrodes with an external tip diameter of 2–4 μm was pulled from 1.5 mm diameter glass capillaries (TW150-4; World Precision Instruments, Inc., Sarasota, FL) with an electrode puller (P-97; Sutter Instrument Co., Novato, CA). After 10 min of equilibration in a measuring solution (0.5 mM KCl, 0.5 mM NaCl, 0.1mM MgCl_2_, 0.2 mM CaCl_2_, and 2.5% sucrose; pH was adjusted to 5.7 with HCl and KOH), we continuously recorded the steady-state fluxes of K^+^ for 10–15 min. Ion fluxes were measured along the callus cell. The software of imFluxes V2.0 (YoungerUSA LLC, Amherst, MA 01002, USA), which is capable of integrating voltage signal, motion control, and image capture simultaneously was used. The net K^+^ fluxes were then calculated using the Mage Flux software developed by Xuyue (http://xuyue.net/mageflux).

### 2.12. Statistical Analysis

The results of growth indexes and net ion fluxes were examined by one-way ANOVA using SPSS21. Significant differences between mean values were determined through Duncan’s multiple-range tests; otherwise, a Student’s *t*-test was indicated. When *p* < 0.05, the differences were considered statistically significant.

## 3. Results

### 3.1. K^+^ Distribution under Normal and Salinized Conditions

**Figure 1:**
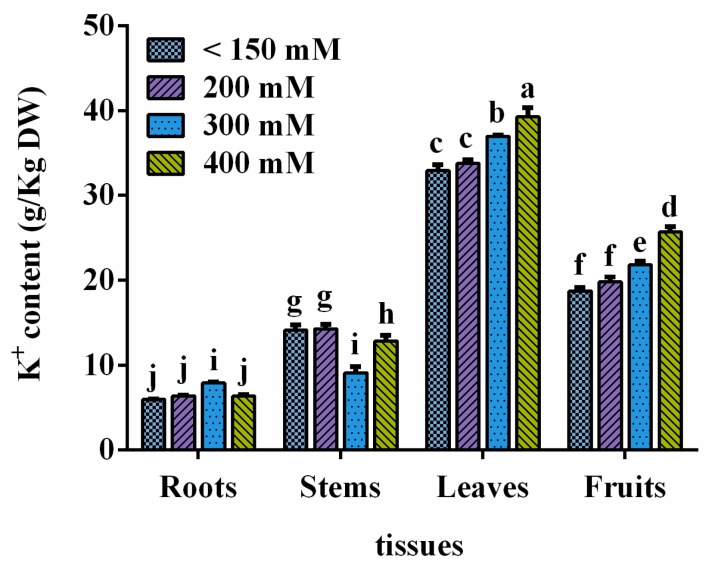
Ion contents of different tissues for *L. ruthenicumin* in different saline soils. DW means dry weight. The mean ± standard error (SE) of five replicates is presented. The lowercase letters on the histogram indicate *p* < 0.05.

K^+^ contents in different tissues were analyzed using three-year-old *L. ruthenicum* trees growing under different salinized conditions. The K^+^ content in the different tissues of *L. ruthenicum* significantly varied. In general, the K^+^ contents in leaf and fruit were significantly higher (*p* < 0.05) than those in the root and stem, and the K^+^ content in leaf was significantly higher than that in fruit (Figure 1). The K^+^ content of trees did not show significant differences between mildly salinized soils and natural soils (which used as a control), indicating that the small increase of salinity did not affect the K^+^ content. The K^+^ contents of the root in moderate soils (about 300 mM NaCl) were significantly higher than those in mildly (about 200 mM NaCl) and severely salinized (about 400 mM NaCl) soils. The opposite results occurred in the stem, where the K^+^ contents in moderately salinized soil were significantly lower than those in mildly and severely salinized soil. The K^+^ content in leaves and fruits significantly increased with salinization, thereby indicating that the edible parts of *L. ruthenicum* could enrich K ions under high salinization conditions.

### 3.2. Cloning, Protein Sequence Alignment, and Phylogenetic Analysis of LrKUP8

**Figure 2:**
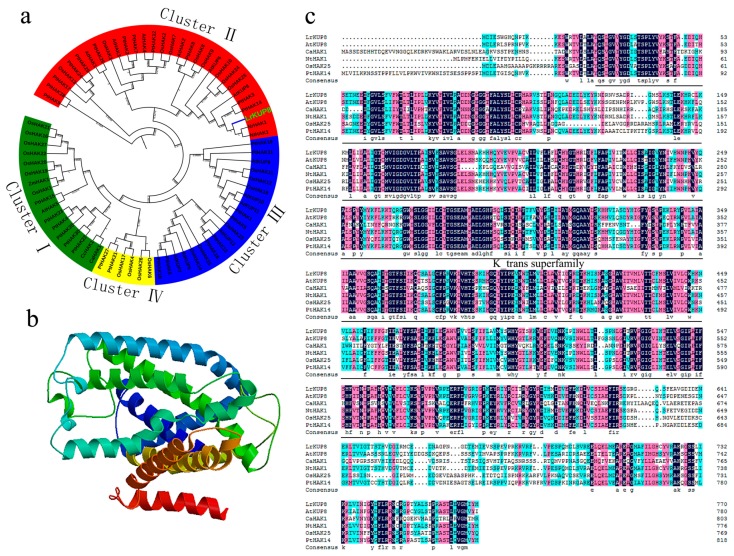
In silico analysis of the KT/KUP/HAK protein. (**a**) The phylogenetic tree using KUP transporters from *A. thaliana*, *Nicotiana tabacum*, *O. sativa*, and *Populus*. The tree topology was constructed using the Clustal W2 (http://www.ebi.ac.uk/Tools/msa/clustalw2/) and MEGA programs. LrKUP8 is indicated by green enlarged font. (**b**) Protein prediction of LrKUP8. (**c**) Multiple alignments of amino-acid sequences of LrKUP8 and other plant KT/KUP/HAK transporters.

According to our previous transcriptome of *L. ruthenicum*, a highly salt-induced gene annotated as a K transporter was isolated from salt-treated calli. The presence of a typical “K_trans” domain (Pfam Profile PF02705) indicates that the isolated gene encodes a putative HAK transporter family member (Figure 2a). The three-dimensional (3D) structure of LrKUP8 suggests that it contains 15 strong transmembrane helices and the typical characteristics of a membrane protein (Figure 2b). BLAST searches against the TAIR database revealed that the deduced amino-acid sequence of this protein is highly similar to that of high-affinity KUP8-like K transporters from Arabidopsis. Thus, this gene was designated as *LrKUP8*. Phylogenetic analyses of *LrKUP8* and the KUP family members from Arabidopsis, rice, and poplar showed that *LrKUP8* is homologous to *NrHAK1*, *NtHAK1*, and *AtKUP8*, and belongs to subgroup II of the KUP family (Figure 2c). These four homologous genes were not characterized.

### 3.3. The Subcellular Localization and Expression Pattern of LrKUP8

**Figure 3:**
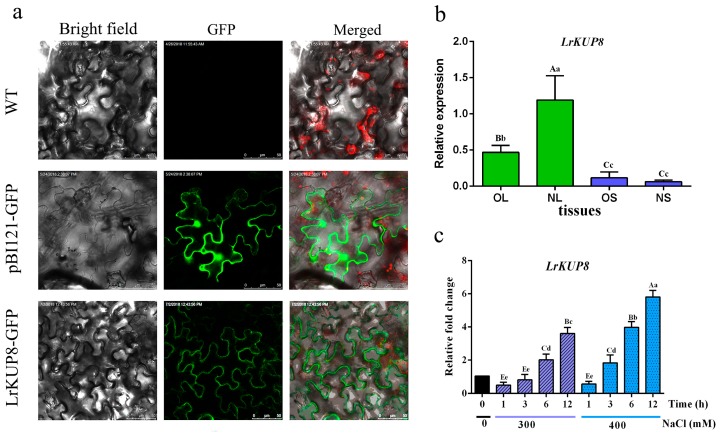
Cellular localization and expression patterns of *LrKUP8*. (**a**) Tobacco epidermal cells were transiently transformed with constructs containing either control (GFP alone) or LrKUP8–GFP fusion plasmid. (**b**) Tissue expression level of *LrKUP8.* OL, NL, OS, and NS indicate old leaves, new leaves, old stems, and new stems, respectively. The relative expression of different developmental tissues was calculated by the 2^−ΔCT^ method. (**c**) *LrKUP8* expression levels in the calli under different saline conditions. The relative fold change data under different salt treatments were analyzed using the 2^−ΔΔCT^ method. The upper- and lowercase letters on the histogram indicate *p* < 0.01 and *p* < 0.05, respectively. The mean ± SE of three replicates is presented.

To test the subcellular localization of LrKUP8, we constructed LrKUP8–GFP fusions driven by the Cauliflower mosaic virus 35S promoter and transiently expressed it into tobacco epidermal cells. The cells transformed with the pBI121::GFP vector were used as controls, and GFP fluorescence was observed mainly in the cytoplasm and nucleus (Figure 3a). The green fluorescence of LrKUP8–GFP was confined to the plasma membrane (PM), thereby indicating that LrKUP8 is mainly targeted to the PM. The subcellular localization result also suggests the potential transport activity of LrKUP8. Ion transporters are often differentially expressed in old and new tissues. Thus, we analyzed the tissue-specific expression of *LrKUP8* by qRT-PCR using a panel of four organ/tissue types from three independent three-year-old *L. ruthenicum* trees. *LrKUP8* was expressed ubiquitously in four testing tissues, including new and old leaves and new and old stems. A significantly higher expression level of *LrKUP8* was observed in the new leaves compared with other tissue types (Figure 3b). According to a preparatory experiment for salt resistance of calli, 300 mM NaCl could be used as a threshold for callus salt treatment. Thus, we examined the gene expression level of *LrKUP8* in 300 mM or higher (400 mM) NaCl treated calli. As a result, the relative expression levels of *LrKUP8* in calli under 300 mM NaCl were stable during the first 6 h but increased dramatically at 12 h. When the NaCl concentration increased to 400 mM, *LrKUP8* expression was induced immediately and continually increased until 12 h (Figure 3c). The expression level of *LrKUP8* under 400 mM NaCl treatment was higher than that under 300 mM NaCl treatment at the specific time point. Our results indicated *LrKUP8* expression could be induced by exposure to salt stress, especially to high salt stress.

### 3.4. Overexpression of LrKUP8 Improved Callus Salt Tolerance

**Figure 4:**
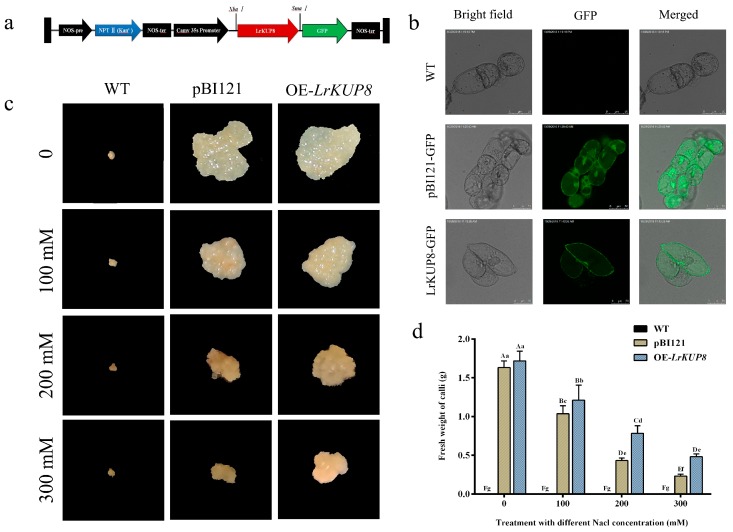
Overexpression of *LrKUP8* confers salt tolerance in transgenic calli. (**a**) Schematic representation of the T-DNA region of pBI121. The red and green boxes indicate *LrKUP8* and *GFP*, respectively. (**b**) GFP detection of vector control and *LrKUP8* overexpressing calli. (**c**) Phenotype test of vector control and OE-*LrKUP8* lines after being grown on medium containing different Na^+^ concentrations and 50 mM kanamycin for 50 days. (**d**) Fresh weight analysis of vector control and OE-*LrKUP8* lines after being grown on medium containing different Na^+^ concentrations for 50 days. The data in (**d**) are shown as means ± SE (*n* = 3). The upper- and lowercase letters on the histogram indicate *p* < 0.01 and *p* < 0.05, respectively.

Expression analysis indicated that *LrKUP8* is strongly induced by salt, and its protein is targeted to the PM. Thus, *LrKUP8* might be involved in ion acquisition under salt treatment. Calli were transformed with the pBI121–LrKUP–GFP recombinant vector to study whether *LrKUP8* regulates salt tolerance, and the pBI121–GFP vector was used as the control (Figure 4a). After screening from a medium with kanamycin, *NPTII*, *LrKUP8-GFP*, and *GFP* were examined by PCR amplifications to guarantee the genome presence of *LrKUP8* (Figure S1, Supplementary Materials). Before the transgenic lines were re-cultured for salt treatment, GFP imaging was also performed to ensure the expression of LrKUP8–GFP protein. As expected, the GFP signal was absent in wide type calli but was observed mainly in cytoplasm and nucleus in the empty vector control (Figure 4). Comparatively, LrKUP8-GFP fluorescence was predominantly localized at the membrane system (Figure 4b), indicating that LrKUP8 expressed normally in transgenic calli. The kanamycin was always used to ensure that all or most of the transgenic callus cells were positive cell clones. In the absence of salinity stress conditions, the vector control and *LrKUP8* overexpressing calli exhibited similar growth rates. The differences appeared at 100 mM NaCl and became highly pronounced at 200 mM NaCl. The 300 mM NaCl treatment considerably delayed the growth of all studied calli (Figure 4c). However, the growth of OE-*LrKUP8* calli under the 300 mM NaCl treatment was better than that of the control calli because the vector control calli almost stopped growing, whereas the transgenic calli continually grew. The wild-type callus cells could not grow because they were not resistant to the aminoglycoside antibiotic kanamycin. The fresh weight analysis also indicated that when the NaCl concentration was 200 mM or higher, the OE-LrKUP8 calli grew considerably faster than the vector control calli (Figure 4d). Therefore, our results indicate that the overexpression of *LrKUP8* improves callus salt tolerance, especially at a high NaCl level.

### 3.5. LrKUP8 Functions in K^+^/Na^+^ Homeostasis through K Accumulation under Salt Stress Condition

**Figure 5:**
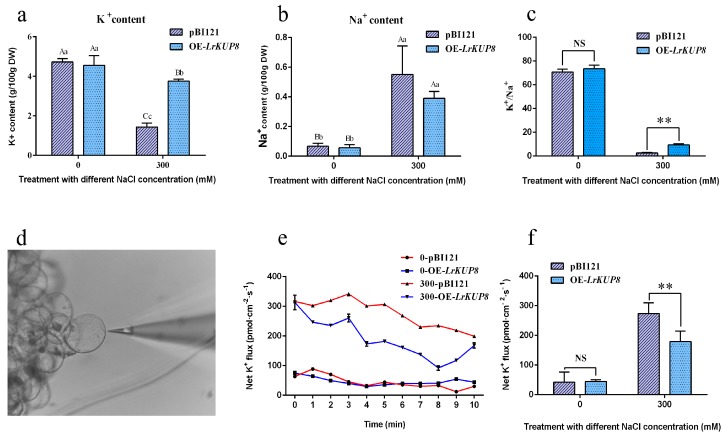
*LrKUP8* reduces K^+^ efflux in calli. (**a**) K^+^ content of *L. ruthenicum* calli with 300 mM NaCl for 50 days expressing *GFP* or *LrKUP8-GFP*. The data is from three samples (*n* = 3). (**b**) Na^+^ content of *L. ruthenicum* calli with 300 mM NaCl for 50 days expressing *GFP* or *LrKUP8-GFP*. The data is from three samples (*n* = 3). (**c**) K^+^/Na^+^ ratios of *L. ruthenicum* calli with 300 mM NaCl for 50 days expressing *GFP* or *LrKUP8-GFP*. The data is from three samples (*n* = 3). (**d**) The measuring position using K^+^-selective microelectrode of NMT. (**e**) K^+^ flux rates measured by NMT/MIFE assays of *L. ruthenicum* calli expressing *GFP* or *LrKUP8-GFP* under control or 300 mM NaCl treatment after 12 h. The data is from six samples (*n* = 6). (**f**) Histogram analyses of K^+^ in *L. ruthenicum* calli expressing *GFP* or *LrKUP8-GFP* under control or 300 mM NaCl treatment after 12 h. The data is from six samples (*n* = 6). The mean ± SE of all replicates is presented. The upper- and lowercase letters on the histogram indicate *p* < 0.01 and *p* < 0.05, respectively. The double asterisks represent *p* < 0.01. NS means not significant.4. Discussion.

We tested the K^+^ and Na^+^ concentrations of OE-*LrKUP8* and vector control calli before and after salt treatment. The 300 mM NaCl treatment for 50 days substantially decreased the K^+^ content but increased the Na^+^ content in both control and transgenic calli. The K^+^ content decreased by 61.7% after salt treatment in the vector control and by 17.1% in OE-*LrKUP8* calli (Figure 5a). Thus, the K^+^ content in OE-*LrKUP8* was significantly higher than that of vector control after 50 days of growth under high salt conditions. The Na^+^ content increased to 5.5 and 3.92 times after salt treatment in the vector control and OE-*LrKUP8* calli, respectively (Figure 5b). As a result, K^+^/Na^+^ ratios in the control and transgenic calli were significantly reduced after salt stress. The OE-*LrKUP8* calli showed a higher K^+^/Na^+^ ratio compared with the vector control (Figure 5c), suggesting that this ratio is affected by the K^+^ translocation because of *LrKUP8* overexpression. Different types of calli grown with or without NaCl treatment were used for K^+^ flux assessments. The glass electrode (2–4 μm aperture), which was filled with liquid electrolyte, was placed at an appropriate distance apart from the calli cell (Figure 5d). All the tested calli exhibited minimal K^+^ efflux in the absence of NaCl stress, and no statistical difference occurred between the two kinds of calli (Figure 5e). We then measured the K^+^ flux responses under 300 mM NaCl conditions. After 12 h of salt treatment, we detected a steady K^+^ efflux in the control and transgenic calli. The efflux rate under salt stress was considerably larger than that in untreated experiments (Figure 5e). Furthermore, the rate of K^+^ efflux in the vector control was remarkably higher than that in transgenic callus lines (Figure 5f).

The use of a transgene is the most commonly applied method for analyzing gene function, but stable genetic transformation approaches are difficult to achieve for certain plants [43]. *L. ruthenicum* is a rare medicinal-and alimental-use species with high saline resistance. Numerous salt response genes were screened from *L. ruthenicum* via the RNA-sequencing method. However, few genes were characterized because of the difficulties or long period required for the genetic transformation of this species. *L. ruthenicum* is a self-incompatible outcrossing species. Hence, all genotypes of cultivars are highly heterozygous. Different genotypes within a cultivar affect the transformation efficiency and potentially the characteristics of transgenic plants. With the increasing interest in breeding and gene mining of *L. ruthenicum*, novel transformation method was developed [44]. These techniques differ in the explant source and procedure and facilitate the studies on *L. ruthenicum*, but they still require refinement to improve efficiency. In the present study, embryogenic calli were induced from *L. ruthenicum* leaves, and genetic transformation was conducted by the *Agrobacterium*-mediated method. DNA examination and GFP imaging showed that transgenic calli were obtained successfully. Using *L. ruthenicum* calli, numerous transgenic plants carrying multiple genes of interest could be produced after rapid characterization.

The accumulation of Na^+^ usually reduces K^+^/Na^+^ homeostasis in the cytosol because Na^+^ can compete with K^+^ for binding sites at the plasma membrane [8,45,46]. The efflux of Na^+^ or the uptake of K^+^ is the main strategy used by plants to maintain the K^+^/Na^+^ ratio [47,48,49,50]. Many studies have shown that the ratio of K^+^/Na^+^ in cells determines the metabolic capacity of cells, rather than the absolute content of Na^+^ or K^+^ [51,52]. In this study, a putative salt-responsive K^+^ transporter designated as *LrKUP8* was cloned and characterized. Our phylogenetic analyses indicated that *LrKUP8* belongs to the cluster II group of the KT/KUP/HAK family. The KUP/HAK/KT family is the earliest, most abundant, and most functional family of potassium ion transporters, and is considered to play a key role in mediating the accumulation of potassium in cells and maintaining the normal growth of plants [53]. This gene is expressed in all tissues of *L. ruthenicum*, which is different from cluster I members that are expressed exclusively in roots. Although several KT/KUP/HAK members were extensively studied, limited studies were conducted on the homologous genes of *LrKUP8*.

The precise function of *LrKUP8* was further characterized by using the callus system. Biomass is frequently used as the main criterion for identifying salinity tolerance in plants because it represents the combined genetic and environmental effects on plant growth [54,55]. Biomass analysis revealed that OE-*LrKUP8* is less sensitive to salt stress. Therefore, *LrKUP8* plays a key role in the response to a highly saline condition. Analysis of the *LrKUP8* expression pattern is critical to explaining its physiological functions in plants. By studying the expression of *LrKUP8* in different tissues, we found that the relative expression of *LrKUP8* was highest in new leaves. Thus, we proposed that in the process of growth and development, plants will preferentially supply K^+^ to the new leaves, and provide K^+^ to the last important tissues, such as old leaves. The K^+^/Na^+^ ratio in both kinds of calli decreased under salt treatment. This reduction is attributed to the reduction in K^+^ uptake and the increase in Na^+^ uptake. The Na^+^ content between the pBI121 control and OE-*LrKUP8* calli showed no significant difference, thereby indicating that the main function of *LrKUP8* may not for Na^+^ transport. The cells of OE-*LrKUP8* calli maintained relatively higher levels of cytoplasmic K^+^ concentration, which means the decreased rate of K^+^ concentration in OE-*LrKUP8* calli was significantly lower than that in the vector control. This resulted a higher K^+^/Na^+^ ratio in OE-*LrKUP8* calli, which is very important for the better performance of them in high salt medium. The function of genes from *KUP/HAK/KT* family is diversity. *PhaHAK5*, which belongs to the Cluster IV group, is one of the routes by which Na^+^ enters cells, indicating some *KUP/HAK/KT* member also function in Na^+^ transporting [56]. The function of *AtHAK5* under low potassium conditions has been thoroughly studied [19,20]. Compared to retrieve K^+^ from low K^+^ condition, the performance of *KUP/HAK/KT* genes under high Na^+^ condition was relatively fewer studied. Our data demonstrated the *LrKUP8* is crucial for K^+^ uptake, especially under high salt condition. We further performed NMT analysis to clarify the contribution of ion fluxes. The NMT is an effective tool for exhibiting the physical salt tolerance mechanisms of plants because it reflects real-time physiological functions of plant roots while maintaining plant root integrity in living plants [41,57,58]. Measurement results showed that K^+^ effluxes were significantly decreased in OE-*LrKUP8* calli compared with the vector control under NaCl stress. Thus, OE-*LrKUP8 L. ruthenicum* calli could maintain high K^+^/Na^+^ in tissues due to their ability to retain K^+^. Considering that one of the key features of plant salt tolerance is the ability of cells to maintain an optimal K^+^/Na^+^ ratio in the cytosol, we propose that the ability of *LrKUP8* to retain K^+^ contributes to the performance for *L. ruthenicum* under saline conditions.

We performed molecular analyses to uncover the function of *LrKUP8* in response to salt stress because salt-induced K^+^ deficiency is the main factor affecting plant growth performance. Examination of K^+^ and Na^+^ accumulation in vector control and OE-*LrKUP8* callus lines confirmed the excellent capacity of *L. ruthenicum* to maintain the K^+^/Na^+^ ratio through effective K^+^ absorption. K^+^ flux measurement via a non-invasive ion-selective microelectrode technique also revealed that OE-*LrKUP8 L. ruthenicum* can maintain K^+^ under NaCl exposure, and this process may be the main mechanism underlying the salt tolerance of *L. ruthenicum*. Our data aid in explaining the K richness of *L. ruthenicum* despite thriving in a natural saline–alkali environment.

## Supplementary Materials

The following are available online at https://www.mdpi.com/2073-4425/10/8/600/s1: Table S1. Primers for *LrKUP8* isolation, *LrKUP8* expression, and OE-*LrKUP8* callus detection; Figure S1. DNA integration analysis to identify *LrKUP8* insertion.

## Abbreviations

DW	dry weight
FW	fresh weight
MDA	malondialdehyde
MS	Murashige and Skoog
NMT	non-invasive micro-test technique
PM	plasma membrane
qPCR	quantitative real-time PCR
