# Supplementary material for: Potassium Transporter LrKUP8 Is Essential for K+ Preservation in Lycium ruthenicum, A Salt-Resistant Desert Shrub
Source: Genes (Basel). 2019 Aug 9;10(8):600. doi: 10.3390/genes10080600 (PMC6723441; doi:10.3390/genes10080600)
Supplement: Supplementary file 1 [file genes-10-00600-s001.zip › supplemental file/Table S1.docx]

Table S1 Primers for *LrKUP8* isolation, *LrKUP8* expression and OE-*LrKUP8* calli detection.

| **Gene name** | **Forward primer ( 5́**′**→3́**′ **)** | **Reverse Primer ( 5́**′**→3́**′ **)** |
| --- | --- | --- |
| *LrKUP8* (isolation) | ATGGATATTGAGAGTTGGGGT | TTATACATGGTAAATCATTCCAACCTC |
| *LrKUP8* (qPCR) | AGGATGGATGTCACTCGGTG | TGCTTGGAAAGATACGCAGC |
| *Actin* (qPCR) | CACCTTCCAACAGATGTGGATT | TCCTGCTCAGAACTCCGACT |
| *Kan* | TGATTGAACAAGATGGATTGC | AGAAGAACTCGTCAAGAAGGC |
| *GFP* | ATGGTGAGCAAGGGCG | CTTGTACAGCTCGTCCATG |
| *LrKUP8-GFP* | GAGGTTGGAATGATTTACCATGT | CTTGTACAGCTCGTCCATG |
