# Supplementary figures and images for: Potassium Transporter LrKUP8 Is Essential for K+ Preservation in Lycium ruthenicum, A Salt-Resistant Desert Shrub
Source: Genes (Basel). 2019 Aug 9;10(8):600. doi: 10.3390/genes10080600 (PMC6723441; doi:10.3390/genes10080600)

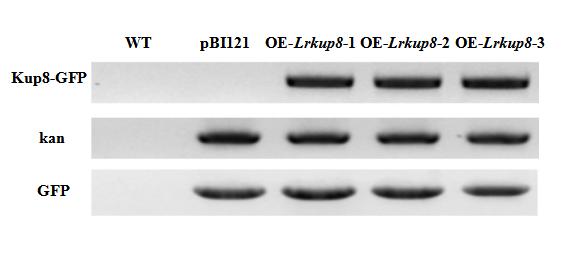

Supplement: Supplementary file 1 [file genes-10-00600-s001.zip › supplemental file/supplemental figure 1.jpg]
